# Effects of N application methods on cotton yield and fertilizer N recovery efficiency in salinity fields with drip irrigation under mulch film using ^15^N tracing technique

**DOI:** 10.3389/fpls.2024.1394285

**Published:** 2024-04-26

**Authors:** Zhen Luo, Wei Tang, Xiaowen Wang, Hequan Lu, Chenyang Li, Jun Liang, Xiangqiang Kong

**Affiliations:** ^1^ Institute of Industrial Crops, Shandong Academy of Agricultural Sciences, Jinan, Shandong, China; ^2^ College of Life Sciences, Shandong Normal University, Jinan, China

**Keywords:** cotton, drip irrigation under mulch film, non-uniform salinity distribution, fertilizer N recovery efficiency, yield, GHNRT

## Abstract

**Introduction:**

Drip irrigation under mulch film promotes a non-uniform salinity distribution in salt fields. The effect of different N application methods on the growth and yield of cotton under drip irrigation under mulch film conditions in eastern coastal saline-alkaline soils in China remain remained unclear.

**Methods:**

A randomized complete block design was used in the experiment. Three N application methods were assigned: N applied under mulch film (low-salinity area; UM), N applied between mulch films (high-salinity area; BM), and half N applied under mulch film and half between mulch films (HUHB).

**Results:**

Plant height, photosynthesis, Chl content, boll load, biomass, boll weight and boll density under UM were all significantly higher than those under the other two treatments. The N absorption of UM was higher than in the other two treatments, which might be attributed to the expression of GHNRT1.5 and GHNRT2.1. The net NO3- influx in the roots in UM increased significantly compared with that in BM. The yield and FNRE of UM were 3.9% and 9.1%, respectively, and were 26.52% and 90.36% higher than under HUHB and BM treatments.

**Discussion:**

UM not only improved cotton yield but also alleviated the pollution of N residue on drip irrigation under mulch film conditions in salt areas.

## Introduction

Cotton (*Gossypium hirustum* L.), a major fiber-producing crop planted globally, is often used as a pioneer crop in saline lands because its salinity threshold is 7.7 dS/m (Maas and Hoffman, 1977; Rocha-Munive et al., 2018; Zhang et al., 2021). However, the negative impacts of salinity on cotton germination, nutrient absorption, photosynthesis and yield reduction have been reported in previous studies (Ashraf and Ahmad, 2000; Sikder et al., 2020). Soil salinity is a major abiotic stressor and a strict factor limiting plant productivity (Wang et al., 2003). Over half of all irrigated soils and about 20% of the world’s cultivated lands are affected by salinity (Arzani, 2008). Therefore, it is of great importance for the sustainable development of the cotton industry to increase cotton yields in saline-alkaline soils.

The response of plants to salt stress varies in different soil environments. The salinity in salt fields is often heterogeneous, and a number of research studies have confirmed that non-uniform salinity in the root zone can alleviate salt injury and promote plant growth, which were mainly attributed to more water absorption by roots from the low salinity zone, increased Na^+^ efflux of the low-salinity roots by the plasma membrane Na^+^/H^+^ antiporter, and decreased Na^+^ concentration in leaves through transporting excessive Na^+^ from leaves to the low-salinity roots (Bazihizina et al., 2009, 2012; Kong et al., 2012, 2016). Our previous studies have shown that nutrient uptake, such as nitrogen uptake, increases on the non-saline root side, and cotton growth, photosynthesis and transpiration are improved under non-uniform salinity compared with that under uniform salinity (Kong et al., 2016, 2017).

Drip irrigation technology under mulch film, which can save water and prevent salt accumulation, succeeded and was popularized extensively in Xinjiang in northwest inland China in 1996 (Zhang et al., 2020). A study on water and salt migration in cotton fields with drip irrigation under mulch film showed that salt migration is mainly divided into the leaching process of salt during irrigation and the redistribution process of salt with water after irrigation (Wang et al., 2000; Lu et al., 2001). Qi et al. (2007) reported that salinity migrated from the soil surface to the lower soil layer during drip irrigation; In addition, the evaporation of soil water was restricted by plastic. Accordingly, a complete desalination region was formed in the root area, which was beneficial for cotton growth and development (Tan et al., 2008; Fu et al., 2013). In other words, the low salinity region was formed around the drip tube under the mulch film and other regions had high salinity (**Supplementary Figure 1**).

Nutrient uptake and plant growth of cotton were inhibited under salinity stress; however, nutrient uptake increased on the non-saline root side under non-uniform salinity compared with that under uniform salinity (Kong et al., 2017). The non-uniform distribution of salinity in salt cotton fields was promoted by drip irrigation under mulch film (Mu et al., 2011). Accordingly, the uptake of N, a vital macronutrient, is increased in saline cotton fields by drip irrigation under mulch film conditions with proper N application methods (Yan et al., 2015; Luo et al., 2018). The enhancement of N supplementation can not only improve the water status of cotton plants but also alleviate salt damage, such as a decline in the generation of lethal oxidative stress biomarkers and an increase in the accumulation of osmolytes (Singh, 2014; Singh et al., 2019; Sikder et al., 2020). Both the northwest inland and eastern coastal saline-alkali land are currently the main cotton-producing regions in China. Due to the different climates, natural conditions and economic development, drip irrigation technology under mulch film has been extensively popularized in the northwest inland but not in the eastern coastal saline-alkali cotton land (Mao, 2013; Zhang et al., 2020). Therefore, we hypothesized that the different N application methods for drip irrigation under mulch film conditions would affect the growth and yield of cotton in eastern coastal saline-alkaline soils in China. The objectives of the present study were to determine: a) the effect of N application methods on cotton growth and yield and b) the effect of N application methods on N uptake and FNRE using a ^15^N tracing technique in saline fields on drip irrigation under mulch film conditions.

## Materials and methods

### Field experiment

#### Experimental sites

**Figure 1:**
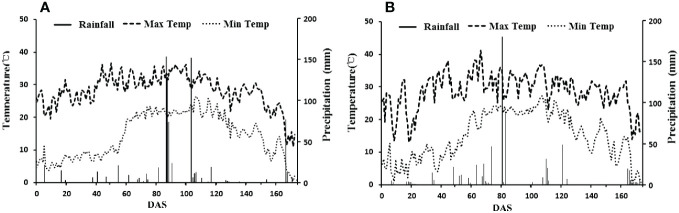
Temperature and precipitation of experimental site in 2021 **(A)** and 2022 **(B)**.

The field experiments were conducted in Wudi county (117·61′ E, 37·74′ N), Shandong, China. The climate in this region is warm, temperate and semi-humid monsoon. The average annual temperature is 12.7°C, and the annual effective accumulated temperature is 4300–4400°C. The annual average precipitation is 564.8 mm, and the annual average humidity is 66%. The annual average evaporation is 1806 mm, and the average annual duration of sunshine is 2632 h, with a 205-day frost-free season for crop growth. Daily weather variables of the experimental site of the growth season in 2021 and 2022 are shown in **Figure 1**. Wudi County is a part of the Yellow River Delta. The soil in this area is coastal saline soil, and the top 20 cm of the soil contains organic matter (9.25 g kg^−1^), alkali hydrolysable N (39.58 mg kg^−1^), available P (12.53 mg kg^−1^), available K (491.31 mg kg^−1^), soil salinity (0.28%) and pH of 8.10.

#### Experimental design and field management

The experiment used a randomized complete block design and each treatment was repeated three times. Three N application methods were assigned as follows: all N applied under the mulch film (UM), all N applied between the mulch films (BM), half N applied under the mulch film and half N applied between the mulch films (HUHB) (**Supplementary Figure 2**). Each plot was 66 m^2^ in area (14.5 m × 4.56 m), consisting of 6 rows with 2 rows under each mulch film, which were equally separated with 76 cm spacing. The drip irrigation tube was placed in the middle of the two rows under the mulch film. Plots with good drainage and irrigation conditions were selected as the experiment sites. The experiment was performed in 2021 and repeated in 2022. The main local cotton cultivar ‘SCRC37’, whose male and female parent both are Asian cotton lines, was cultivated by the cotton breeding team of institute of industrial crops, Shandong academy of agricultural sciences and used in this experiment. The cotton was sown on 17 April 2021 and 20 April 2022 and harvested on 8 October 2021 and 10 October 2022. The planting density was 9.00 × 10^4^ plants ha^-2^. Due to the rainy season in July and August in the local area, cotton is usually irrigated three or four times during the whole growing season. In the present study, the water content of the soil was monitored by soil moisture detection system DX5398 (Zhongnong Zhizao, China), irrigation occurred on 18 April, 3 June, 2 July and 21 August in 2021 and 21 April, 5 June and 25 August in 2022 when the soil moisture is lower than 60% of soil water holding capacity. The volume of drip irrigation was 450 m^3^ ha^−1^. Throughout the growing season, about 80% of the soil surface was covered with mulch film. Each plot was fertilized with 150 kg ha^−1^ of N (in the form of urea), 75 kg ha^−1^ of P_2_O_5_ and 60 kg ha^−1^ of K_2_O. All fertilizers were applied as basal fertilizers when sowing.

To prevent surface runoff pollution and lateral infiltration, ^15^N-labeled urea was applied in the microplots (1 × 1 m), which were enclosed with hard plastic squares. The height of the hard plastic square was 0.45 m, of which 0.40 cm was buried in the soil and 0.05 m of the square remained above the soil surface. Three microplots were set aside in each plot. Urea, used in the experiment, was enriched with 10.2% atom ^15^N (provided by the Institute of Chemical Industry, Shanghai, China). To ensure normal growth of the cotton, all experimental plots were managed uniformly according to local agronomic practices.

### Data collection

Data were collected for net photosynthetic (Pn) rate, chlorophyll (Chl) content, boll load, biological yield, seedcotton yield, harvest index, N uptake in vegetative organs and reproductive organs, ^15^N uptake in vegetative organs, and FNRE.

### Net Pn rate, Chl content and boll load

The net Pn rate of the 3rd youngest fully expanded leaf from the main stem terminal was determined from 09:00 to 11:00 h on cloudless days when ambient photosynthetic photon flux density exceeded 1500 μM·m^−2^·s^−1^, using an LI-6800 portable photosynthesis system (Li-Cor, USA). In the meantime, the Chl content of the 3rd leaf was measured by SPAD 502DL plus (Spectrum, USA). The mean value was calculated from three plants per replicate. The leaf area was determined using an LI-3100 leaf area meter (Li-Cor, USA), and the leaf area index (LAI) was determined according to the ground area. All reproductive organs (squares, flowers, green and mature bolls) were dried at 105°C for 30 min, dried at 80°C for 48 h to a stable weight and weighed. The boll load (dry weight of reproductive organs per unit leaf area) was then determined.

### Biological yield, seedcotton yield, yield components and harvest index

To determine boll weight, 50 bolls were randomly selected and harvested in the four central rows from each plot. After 10 days of sun drying, the boll weight was determined after the seedcotton presented the same weight in two successive weights. Seedcotton yield (kg·ha^−1^), the average boll weight and boll density were determined for each plot. Seedcotton in the microplots was harvested and stored separately. Ten plants were sampled randomly out of microplot from each plot, pulled out naturally, and divided into leaves (previous fallen leaves included), stems and roots after harvest. All plant samples were dried at 105°C for 30 min, dried at 80°C for 48 h to a stable weight and weighed. Plant biomass, dry matter distribution and harvest index (seedcotton yield/biological yield) were then determined (Bange and Milroy, 2004; Luo et al., 2018).

### Soil salinity, N uptake, ^15^N accumulation and FNRE

Soil samples in each plot under mulch film or between mulch films were collected from 0 to 20 cm depth using a 5 cm i.d. auger at 0, 30, 60, 90, 120 and 150 days after sowing (DAS). The soil salinity of the water-saturated soil paste extract was measured using an electrical conductivity meter, S230 (Mettler Toledo, Switzerland).

Two plants from each microplot were selected randomly, pulled out naturally, and divided into leaves (including previously fallen leaves), stems, roots, boll shells and seedcotton. Each part of the plant from the microplots was dried at 105°C for 30 min and then dried at 80°C to a stable weight before measuring the dry weight. All materials were ground and then sieved through a 0.25-mm sieve, and 5 g powder was collected randomly for total N and ^15^N isotope analysis. The total N concentration and ^15^N abundance were analyzed using an isotope mass spectrometer (Delta V Advantage, Thermo Fisher, Waltham, MA).

The amount of plant N derived from fertilizer (Ndff) was calculated following Equation 1 (atom% ^15^N of naturally occurring N is 0.3663%):


(1) Ndff=(the atom%N15 of crops ample–0.3663)/(the atom%N15 ofN15-labeled urea–0.3663)×100%

^15^N accumulation in different organs of cotton plants and FNRE was calculated following Equations 2 and 3:


(2) N15 accumulation=total N accumulation in cotton plant×Ndff

(3) FNRE=N15 accumulation in cotton plant / Nappl.×100%

### Laboratory experiment

#### Experimental treatments

To determine the expression of *GHNRT* genes and the flow rate of NO_3_- in cotton roots, the non-uniform distribution of salinity was simulated using a longitudinal salt difference distribution device (**Supplementary Figures 3** and **4**). The device consisted of the upper and lower parts. To more accurately simulate the non-uniform salinity distribution of drip irrigation under mulch film in the saline field, treatments with the same NaCl content (1.5 g·kg^−1^) in the soil of the two parts represented the uniform distribution salinity stress treatments, denoted as 1.5/1.5. The treatment with the different NaCl content (1.5 or 3.0 g·kg^−1^) in the two parts represented the non-uniform distribution salinity stress, denoted as 1.5/3.0. As the control, the two parts of the treatment were free of NaCl.

#### Real-time PCR analysis

The expression of *GHNRT* genes was determined using quantitative real-time PCR (RT-qPCR). After 7 days of treatment, root tissues from both sides of all four treatments were harvested and extracted for total RNA using TRIzol reagent (Invitrogen). Primer Premier 5.0 (Premier Biosoft International) was used to design the gene-specific primers according to the gene sequences and was then synthesized commercially (Shanghai Sangon Biological Engineering Technology & Services). The primers and gene identifiers are listed in **Supplementary Table 1**.

The PCR program was performed according to Kong et al. (2016). Each treatment was analyzed with three biological replicates. The expression level of *actin* was used to normalize the RT-qPCR results relative to the NaCl-free control. SAS software was used to calculate the Pearson correlation coefficients of the expression patterns of the selected genes.

#### Measurements of net NO_3_- flux with NMT

Non-Invasive Micro-Test Technology (NMT) [NMT System BIOIM; Younger USA, LLC.] was used to measure the net flux of NO_3_- according to Kong et al. (2012). After 7 days of treatment, 2-3 cm root segments from the apices were cut down and washed with redistilled water and then incubated in the measuring solution immediately to equilibrate for 30 min, and then put into the measuring chamber containing 10-15 ml fresh measuring solution. The measuring solution consisted of 0.1 mM NH_4_NO_3_, 0.1 mM CaCl_2_ and 0.3 mM MES, and the pH was adjusted to 6.5 with choline and HCl. A site 5 mm from the root apex was used to measure the net flux of NO_3_-. Two-dimensional ionic fluxes were calculated using MageFlux developed by Yue Xu (http://xuyue.net/mageflux).

### Statistical analysis

Analysis of variance (ANOVA) was performed using the randomized complete block design function of the DPS Data Processing System (Version 11.50; Tang and Feng, 2002). The combined data showed that there were no interactions with the years. Therefore, the data were polled across the two years. Means were separated using Duncan’s multiple range tests at P< 0.05 for significant differences.

## Results

### Changes in soil salinity during the growing season

**Figure 2:**
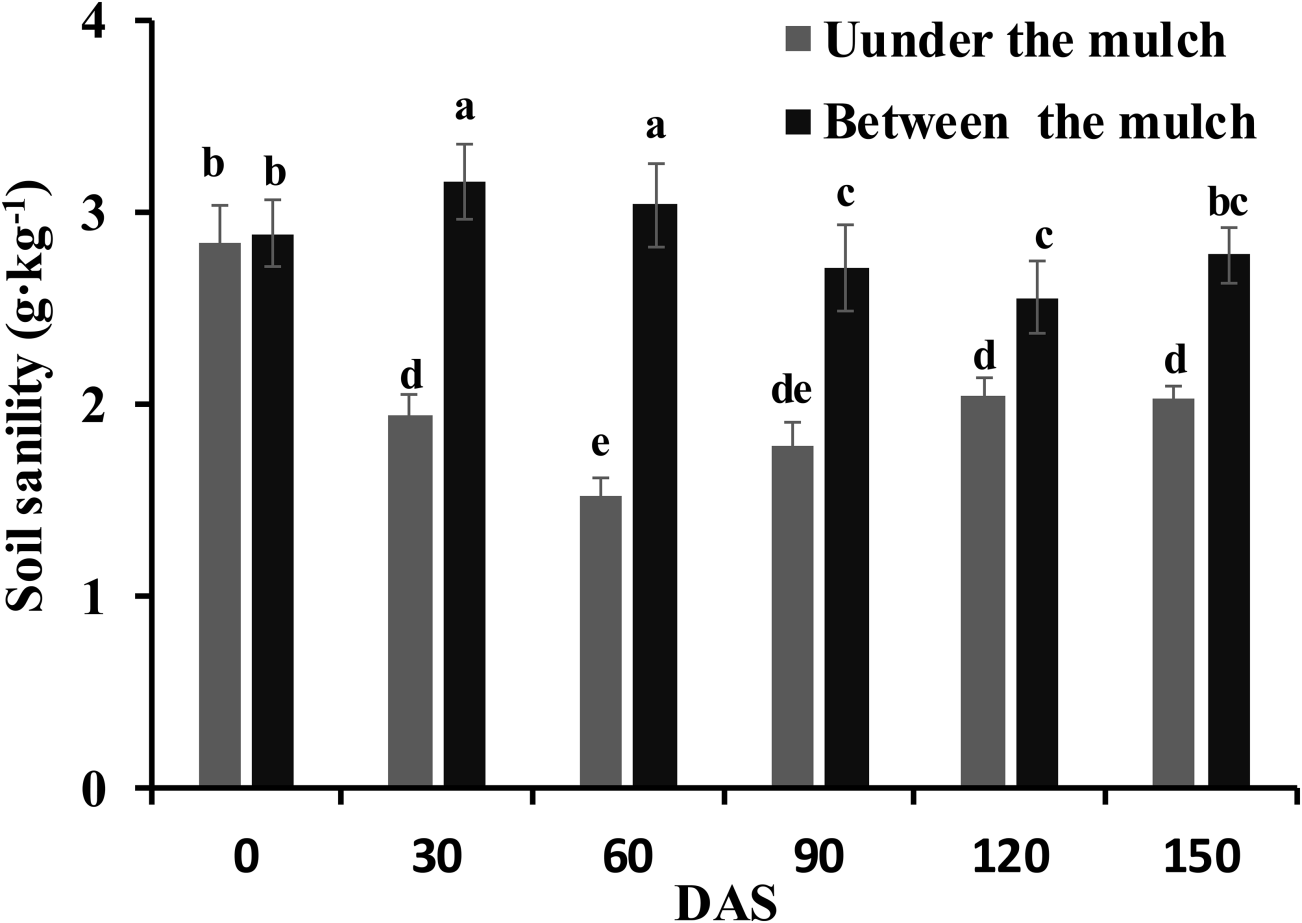
Changes in the soil salinity at 0, 30, 60, 90, 120 and 150 days after sowing. Different letters in the figure indicate statistically significant differences (P< 0.05) after ANOVA and the LSD tests.

The soil salinity at 0, 30, 60, 90, 120 and 150 DAS are shown in **Figure 2**. There were no differences in the soil salinity under mulch film or between mulch films among the different N application methods. Therefore, the data on the soil salinity under mulch film or between mulch films were an average of the three application methods. The soil salinity under mulch film first decreased and then increased and ranged from 1.5 to 2.0 g·kg^−1^ from 30 to 150 DAS. In contrast, the soil salinity between mulch films first increased and then decreased and ranged from 2.6 to 3.2 g·kg^−1^ from 30 to 150 DAS. The soil salinity between mulch films was 62.1%, 98.7%, 52.2%, 24.9% and 37.0% higher than that under mulch film on 30, 60, 90, 120 and 150 DAS, respectively.

### Effect of different N application methods on plant growth

**Figure 3:**
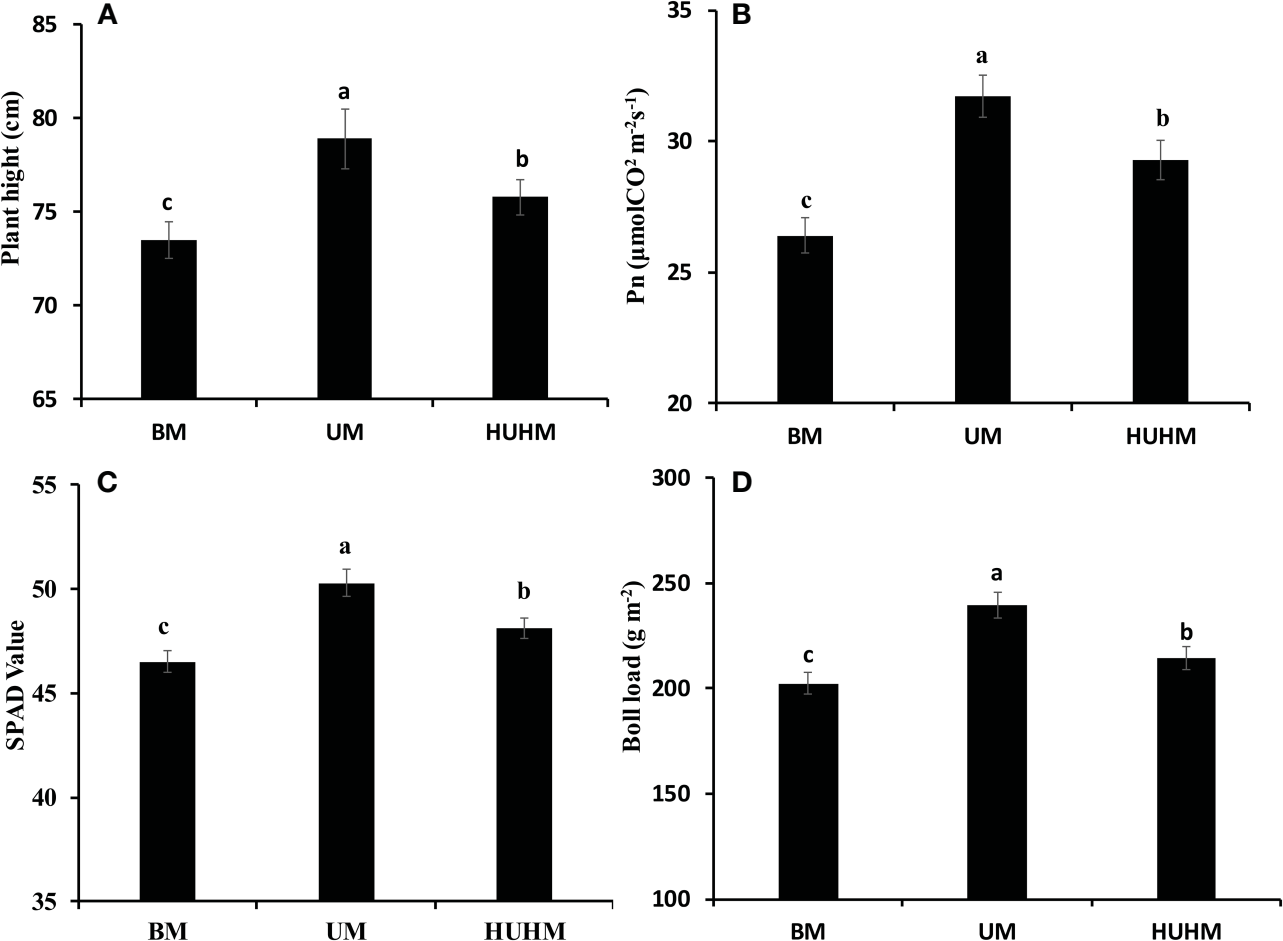
Changes in plant height **(A)**, Pn **(B)**, SPAD value **(C)** and Boll load **(D)** of cotton under different N application methods on drip irrigation under mulch film condition in salt field. Different letters in **(A–D)** indicate statistically significant differences (P< 0.05) after ANOVA and the LSD tests.

The effects of different N application methods on plant growth at the peak bolling stage are shown in **Figure 3**. The highest plant height, net Pn rate, Chl content and boll load were found under UM treatment. The lowest plant height, Pn rate, Chl content and boll load were found under BM treatment. The plant height, Pn rate, Chl content and boll load under HUHB were between that under UM and BM treatment. The plant height, Pn rate, Chl content and boll load under UM were 7.3%, 19.8%, 8.2% and 18.2% higher than that under BM treatment.

### Effect of different N application methods on dry matter and N accumulation

**Figure 4:**
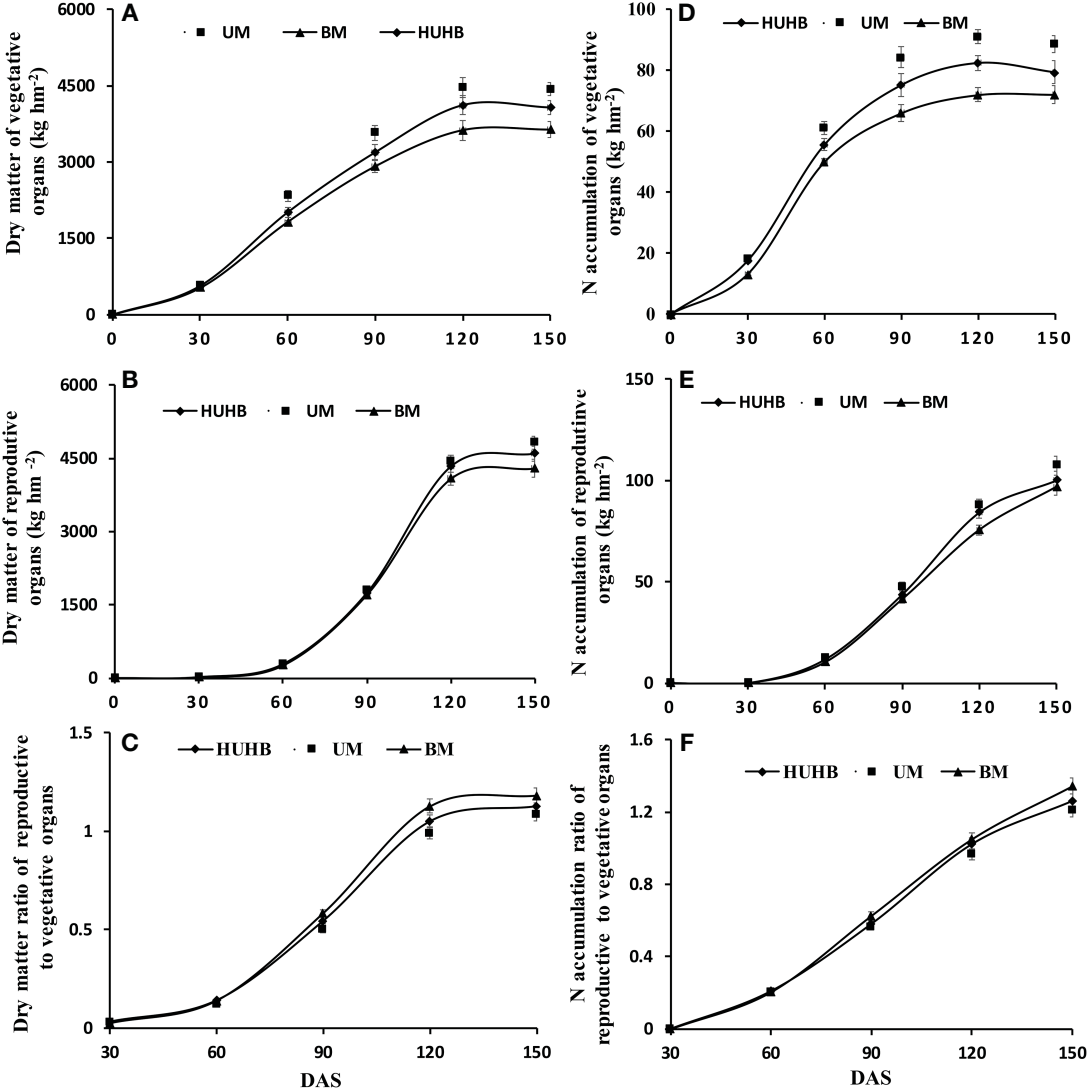
Changes in dry matter, N accumulation and dry matter ratio of reproductive to vegetative organs of cotton under different N application methods on drip irrigation under mulch film condition in salt field. Dry matter of vegetative organs **(A)** and reproductive organs **(B)**, the dry matter ratio of reproductive to vegetative organs **(C)**, N accumulation of vegetative organs **(D)** and reproductive organs **(E)**, the N accumulation ratio of reproductive to vegetative organs **(F)**.

Dry matter and N accumulation in vegetative organs and reproductive organs were measured on 0, 30, 60, 90, 120 and 150 DAS (**Figure 4**). There was a rapid accumulation period of dry matter in the vegetative organs from 30 to 120 DAS (**Figure 4A**). The dry matter accumulation in the vegetative organs of UM was significantly higher than that of HUHB and BM from 60 to 150 DAS. Similarly, dry matter accumulation in reproductive organs entered a rapid accumulation period 60 DAS (**Figure 4B**). However, there were no significant differences among the three treatments until 120 DAS. The dry matter accumulation in the reproductive organs of UM increased 4.9% and 12.3% compared with that of HUHB and BM on 150 DAS, respectively. The dry matter ratio of reproductive to vegetative organs increased rapidly from 60 to 120 DAS (**Figure 4C**). The dry matter ratio of the reproductive to vegetative organs of BM treatment increased 4.7% and 8.6% compared with that of HUHB and UM treatments on 150 DAS, respectively.

The curve of N accumulation was similar to that of dry matter accumulation in cotton plants (**Figure 4A, B, D, E**). N accumulation in the vegetative organs of UM and HUHB was significantly higher than that of BM from 30 to 150 DAS (**Figure 4D**). N accumulation in vegetative organs of UM was 12.1%, 10.7% and 11.8% higher than that of HUHB on 90, 120 and 150 DAS, respectively. N accumulation in reproductive organs maintained a higher accumulation speed from 60 to 150 DAS (**Figure 4E**). N accumulation in the reproductive organs of UM increased 7.4% and 11.0% more than that of HUHB and BM on 150 DAS, respectively. The N accumulation ratio of reproductive to vegetative organs maintained a high increase speed from 30 to 150 DAS (**Figure 4F**). The N accumulation ratio of reproductive to vegetative organs of BM treatment increased 6.5% and 10.9% compared with that of HUHB and UM treatment on 150 DAS, respectively.

### Effect of different N application methods on Ndff and ^15^N accumulation

The Ndff and ^15^N accumulation effect mediated by different N application methods are shown in **Table 1**. The highest Ndff in roots, stems, leaves, boll shells and seedcotton was found under UM treatment, and the lowest Ndff in roots, stems, leaves, boll shells and seedcotton was found under BM treatment. Ndff in roots, stems, leaves, boll shells and seedcotton increased 53.85%, 50.00%, 57.69%, 54.17% and 50.00% under UM treatment compared with BM treatment. Similarly, the highest ^15^N accumulation in roots, stems, leaves, boll shells and seedcotton was found under UM treatment, followed by HUHB and BM. ^15^N accumulation in roots, stems, leaves, boll shells and seedcotton were 95.76%, 65.09%, 80.70%, 92.49% and 105.78% higher, respectively, under UM treatment than under BM treatment.

**Table 1:** Changes in Ndff and ^15^N accumulation of cotton under different N application methods on drip irrigation under mulch film condition in salt field.

	Treatment	Root	Stem	Leaf	Boll shell	Seedcotton
Ndff	HUHB^*^	0.35b^**^	0.34b	0.34b	0.33b	0.34b
	UM	0.40a	0.42a	0.41a	0.37a	0.39a
	BM	0.26c	0.28c	0.26c	0.24c	0.26c
^15^N accumulation (g m^-2^)	HUHB	13.3b	68.5b	174.4b	91.3b	246.0b
	UM	17.0a	85.5a	223.8a	113.4a	311.3a
	BM	08.7c	51.8c	123.9c	58.9c	151.3c

^*^HUHB means half N applied under the mulch film (low salinity area) and half N applied between the mulch films (high salinity area); UM means all N applied under the mulch film (low salinity area); BM means all N applied between the mulch films (high salinity area).

^**^Means within a column followed by same letters are not significantly different at p< 0.05 after ANOVA and the LSD tests.

### Effect of different N application methods on yield, harvest index and FNRE

The yield, yield components, harvest index and FNRE under different N application methods are shown in **Table 2**. The highest yield and biological yield were found under UM treatment, followed by HUHB and BM treatments. The yield of the UM treatment was 3.9% and 9.1% higher than that of the HUHB and BM treatments, respectively. The biomass of the UM treatment was 6.4% and 16.3% higher than that of the HUHB and BM treatments, respectively. Similarly, the highest boll density and boll weight were found under UM treatment. Boll density and boll weight increased by 2.3% and 6.9% under UM treatment compared with that under BM treatment. However, the highest harvest index was found under BM treatment, and there was no difference between HUHB and UM treatments. The highest FNRE was found under UM treatment. FNRE under UM treatment was 26.52% and 90.32% higher than that under HUHB and BM treatments, respectively.

**Table 2:** Changes in yield, yield components, harvest index and FNRE of cotton under different N application methods on drip irrigation under mulch film condition in salt field.

Treatments	Boll density	Boll weight	Yield (kg ha^-2^)	Biomass (kg ha^-2^)	Harvest index	FNRE
HUHB^*^	602ab^**^	4.87b	2932.81a	8668.98b	0.335b	39.57b
UM	612a	4.99a	3047.15a	9227.10a	0.330b	50.67a
BM	598b	4.67c	2792.38b	7932.14c	0.344a	26.31c

*HUHB means half N applied under the mulch film (low salinity area) and half N applied between the mulch films (high salinity area); UM means all N applied under the mulch film (low salinity area); BM means all N applied between the mulch films (high salinity area).

^**^Means within a column followed by same letters are not significantly different at p< 0.05 after ANOVA and the LSD tests.

### Effect of non-uniform root zone salinity on the expression level of the GHNRT gene and NO_3_- flux

**Figure 5:**
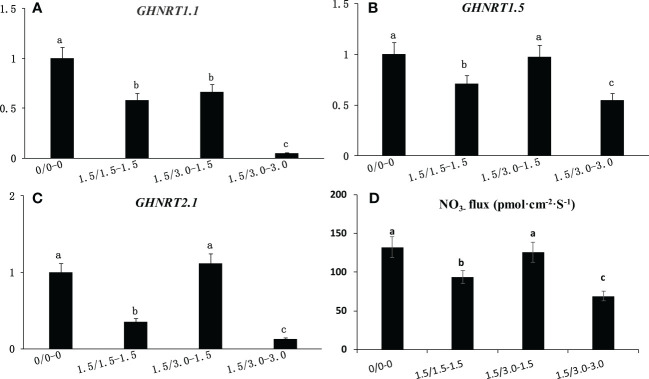
Changes in the expression level of *GHNRT1.1* **(A)**, *GHNRT1.5* **(B)** and *GHNRT2.1* **(C)** and NO_3-_ flux **(D)** in cotton root under non-uniform root zone salinity condition. Different letters in **(A–D)** indicate statistically significant differences (P< 0.05) after ANOVA and the LSD tests.

The expression levels of *GHNRT1.1*, *GHNRT1.5* and *GHNRT2.1* in cotton roots of the laboratory experiment were measured 7 days after treatment (**Figures 5A-C**). The expression level of the three *GHNRT*s decreased under uniform salt stress. The expression of *GHNRT1.1*, *GHNRT1.5* and *GHNRT2.1* under 1.5/1.5 treatment decreased 41.6%, 28.9% and 65.0%, respectively, compared with that under the control (0/0). The expression levels of *GHNRT1.5* and *GHNRT2.1* in the roots from the low salinity area of the non-uniform salt treatment (1.5/3.0-1.5) were comparable with those of the control (0/0). The expression levels of *GHNRT1.5* and *GHNRT2.1* of the roots in the low salinity area of the non-uniform salt treatment (1.5/3.0-1.5) were 102% and 467% higher than that of the uniform salt treatment (1.5/1.5).

NO_3_- flux was also inhibited by salt stress (**Figure 5D**). The NO_3_- flux under 1.5/1.5 treatment decreased 28.7% compared with that under the control (0/0). However, the NO_3_- flux of the roots in the low salinity area of the non-uniform salt treatment (1.5/3.0-1.5) was comparable with that of the control (0/0) and was 34.0% higher than that of the uniform salt treatment (1.5/1.5).

## Discussion

Nutrient uptake decreases drastically under salinity stress, which may inhibit nutrient migration and ultimately lead to yield reduction (Pessarakli, 2001). Various technical means have been used to alleviate salt damage to crop growth (Guo and Lu, 2020). The results of our previous studies indicated that nutrient uptake increases in the low-saline root side under a non-uniform salinity distribution (Kong et al., 2017). Moreover, a non-uniform salinity distribution is formed when using drip irrigation under mulch film (Qi et al., 2007; Mu et al., 2011; Fu et al., 2013). To determine the effects of different N application methods on cotton, plant growth, cotton yield, nitrogen uptake and FNRE were measured using drip irrigation under mulch film conditions in salinity cotton fields with the ^15^N tracing technique.

### Effect of drip irrigation under mulch film on the distribution of soil salinity

Water is the solvent and carrier of fertilizer, salt and other particles; for this reason, salt follows the water in the soil. Previous research has shown that salt accumulated in the regions between the mulch films and to the lower soil layer (Tan et al., 2008; Fu et al., 2013) on drip irrigation under mulch film conditions. The soil salinity under the mulch films was significantly lower than that between the mulch films, especially in the 60 DAS (seedling stage) in the present study (**Figure 2**). Although the rainy season arrived at 80 DAS and the distribution of salt in the soil was affected by rainfall, the soil salinity under the mulch films was significantly lower than that between the mulch films until 150 DAS. The soil salinity was redistributed and the root regions under the mulch films were desalinated to low salinity areas, while the regions between the films changed to high salinity areas. Another reason might be about 80% of the soil surface was covered with mulch film throughout the growing season, and then the water in the soil only evaporated out between membranes, and the salt followed the water moved to the soil between membranes during the process of evaporating. Therefore, the non-uniform salinity distribution in the cotton root area was maintained throughout the whole growing season in the present study.

### Effects of different application methods on the growth of cotton plants

Salt stress can cause many adverse physiological and biochemical effects in cotton plants (Munawar et al., 2021), such as plant height, fresh and dry weights, leaf area, Pn and yield were decreased significantly by salinity stress (Loka et al., 2011). The decrease in photosynthesis was attributed to the reduction of total Chl content and the distortion of Chl ultrastructure under salinity stress (Meng et al., 2011). However, cotton plant growth improved under non-uniform salinity compared with that under the uniform treatment (Kong et al., 2012). N uptake increased significantly under the non-uniform treatment might be attribute to the expression of nutrient transport related genes in the non-saline root side under the non-uniform treatment was higher than that in either root side under the uniform treatment (Kong et al., 2017). In fact, recent studies have shown that increasing N supply could lessen the salinity stress of *Brassica* genotypes (Siddiqui et al., 2010), tomato plants (Singh et al., 2019), wheat seedlings (Ahanger et al., 2019) and cotton plants (Sikder et al., 2020). In the present study, the plant height, Pn, Chl content and boll load under UM treatment were significantly higher than that under BM and HUHB treatments (**Figure 3**). The UM treatment alleviated the salt damage to the cotton plant compared with the BM and HUHB treatments. Accordingly, the alleviated salinity stress under the UM treatment might be attributed to the increased N uptake from the low salinity area, where more available N was applied, compared with the other two treatments.

### Effect of different N application methods on dry matter and N accumulation

Higher dry matter accumulation guarantees a higher cotton yield. It was conducive to maintaining a balance between vegetative and reproductive growth and establishing a reasonable population basis for a high cotton yield so that the dry matter of the population was maintained in an appropriate range (Ye et al., 2004; Zhu et al., 2011). However, the dry matter of cotton decreased significantly under salt stress (Luo et al., 2015; Guo et al., 2022). The leaf and root dry weights decreased gradually as the NaCl concentration increased (Zhang et al., 2014). Although salt stress reduces the accumulation of dry matter in cotton, it might be more conducive to the transport of nutrients to the reproductive organs and increase the dry matter ratio of reproductive to vegetative organs (Feng et al., 2022). In the present study, dry matter accumulation in the vegetative and reproductive organs of UM was significantly higher than that of BM and HUHB (**Figures 4A, B**). The dry matter ratio of the reproductive to vegetative organs of UM obviously decreased compared with that of BM and HUHB (**Figure 4C**) after 120 DAS. The results indicated that UM treatment could alleviate salinity stress in cotton.

N uptake is the basis for dry matter accumulation in cotton (Yin et al., 2016). Many studies have demonstrated a linear positive correlation between dry matter and N accumulation in cotton (Xue et al., 2006; Zheng et al., 2016). The present study indicated that the trend of dry matter accumulation was consistent with N accumulation in cotton across different N application methods using drip irrigation under mulch film in a saline field (**Figures 4A–D**). Although N accumulation in cotton was inhibited by salinity stress (Ma et al., 2013; Luo et al., 2015), the N uptake in the non-saline root side increased significantly under non-uniform salinity conditions in our previous study, which was simulated using a split-root system (Kong et al., 2017). In the present study, non-uniform salinity conditions were formed in the cotton field using drip irrigation under film conditions. The N accumulation of the treatment that N applied in the low-salinity regions (UM) was significantly higher than that of the other two treatments (**Figures 4D, E**). The results indicate that the increased N accumulation of UM treatment might be attributed to the increased N uptake from the low-salinity regions. N accumulation in different cotton organs has been affected by many management practices, such as N application rates, soil and environmental conditions (Shah et al., 2022). The transport of N to reproductive organs was inhibited, and the N accumulation ratio of reproductive to vegetative organs decreased with excessive N application rates (Ma et al., 2013; Luo et al., 2022). Similar results were found in the present study (**Figure 4F**). This further indicates that the effect of increasing the N fertilizer rate can be achieved by changing N application methods using drip irrigation under mulch film in the saline field.

### Effect of different N application methods on Ndff, ^15^N accumulation, FNRE and yield

The N uptake from different sources and the distribution of fertilizer N in different parts of crops can be directly detected by ^15^N tracer technology. Our previous study showed that the Ndff increased with an increase in the N application rate (Luo et al., 2022). In the present study, Ndff and ^15^N accumulation in the cotton plants of the UM treatment were significantly higher than that of the other two treatments (**Table 1**). This indicates that N applied under mulch film (low-salinity area) could increase fertilizer N uptake of the cotton plant in the saline field. The fate of fertilizer N applied to the field includes uptake by crops, left in the soil, or lost in various methods, such as denitrification, runoff, volatilization and leaching. FNRE is affected by the N application method, soil fertility and climate conditions (Fritschi et al., 2004; Roberts et al., 2016). The FNRE of UM treatment was 26.52% and 90.36% higher than that of HUHB and BM treatment in the present study, respectively (**Table 2**). This might be the result of increased Ndff and ^15^N accumulation in cotton plants from the UM treatment. A higher FNRE means a lower N loss and N left in the soil. Consequently, N applied under mulch film in the saline field would alleviate N residue pollution and be beneficial to environmental sustainability.

Boll weight and boll density are two major parameters that contribute to yield. Cotton yield decreased under salinity stress should be attributed to the reduction of boll weight and density with an increase in salinity (Sharif et al., 2019). In the present study, both the highest boll weight and density were found under UM treatment. This further confirmed that UM treatment could alleviate salinity stress using drip irrigation under mulch film in saline fields. High cotton yield depends on adequate total biomass and appropriate harvest index (Ma et al., 2013). In the present study, the lowest harvest index was found under the UM treatment; however, due to the highest total biomass, the highest yield was found under the UM treatment. This showed that total matter production and the balance between vegetative and reproductive growth synergistically determine cotton yield under salinity stress.

### Expression level of the GHNRT gene and NO_3_- flux

Our previous study indicated that the expression of nitrate uptake-related genes (*NRT*) and the net NO_3_- influx on the non-saline root side increased significantly compared with those on the high-saline root side under non-uniform salinity and either root side under uniform salinity (Kong et al., 2017). To more accurately simulate the non-uniform salinity distribution using drip irrigation under mulch film in the saline field, the low-salinity area and the high-salinity area were set at 0.15% and 0.3% with a longitudinal salinity difference distribution device in the lab in the present study. The expression of *GHNRT1.5* and *GHNRT2.1*, as well as the NO_3_- influx in the roots of the low-salinity area, were significantly higher than those of the high-salinity area. This indicates that the increased N accumulation in the cotton plants of the UM treatment might be attributed to the increase in the N absorption from the low-salinity area.

## Conclusion

The non-uniform salinity distribution was formed by the technology of drip irrigation under a mulch film in the eastern coastal saline-alkali cotton field in China. The soil salinity under the mulch films was significantly lower than that between the mulch films throughout the growing season. In the laboratory experiment, the expression of *GHNRT1.5* and *GHNRT2.1* and the net NO_3_- influx in the roots of the low-saline area were significantly higher than those in the high-saline area under non-uniform salinity and roots in either area under uniform salinity. More N was absorbed under the UM treatment than under the other two treatments. Unsurprisingly, the increased N accumulation under the UM treatment alleviates the salt damage of the cotton plant. The plant height, Pn, Chl content, boll load, dry matter accumulation, boll weight and density under UM treatment were significantly higher than those under BM and HUHB treatments. The yield and FNRE of the UM treatment were 3.9% and 9.1%, respectively, and were 26.52% and 90.36% higher than under the HUHB and BM treatments. Accordingly, N applied under mulch film (low-salinity area) not only increased yield but also reduced the environmental pollution caused by nitrogen residue, which would be beneficial to sustainable cotton production in saline-alkaline soils.

## Data availability statement

The original contributions presented in the study are included in the article/**Supplementary Material**. Further inquiries can be directed to the corresponding authors.

## Ethics statement

The authors declare that the collection of plant material complies with Chinese and international guidelines and legislation.

## Author contributions

ZL: Writing – review & editing, Writing – original draft, Supervision, Project administration, Funding acquisition, Data curation. WT: Writing – review & editing, Resources, Methodology, Formal analysis, Conceptualization. XW: Writing – review & editing, Software, Methodology, Investigation, Formal analysis, Data curation. HL: Writing – review & editing, Software, Formal analysis. CL: Writing – review & editing, Methodology, Investigation, Data curation. JL: Writing – review & editing, Methodology, Investigation. XK: Writing – review & editing, Supervision, Project administration, Funding acquisition.

## References

[B1] Ahanger M. A. Qin C. Begum N. Maodong Q. Dong X. X. El-Esawi M.. (2019). Nitrogen availability prevents oxidative effects of salinity on wheat growth and photosynthesis by up-regulating the antioxidants and osmolytes metabolism, and secondary metabolite accumulation. BMC Plant Biol. 19, 479. doi: 10.1186/s12870-019-2085-3 31703619 PMC6839093

[B2] Arzani A. (2008). Improving salinity tolerance in crop plants: a biotechnological view. *In Vitro* Cellular and Developmental Biology-animal. Plant: J. Tissue Culture Assoc. 44, 373–383. doi: 10.1007/s11627-008-9157-7

[B3] Ashraf M. Ahmad S. (2000). Influence of sodium chloride on ion accumulation, yield components and fibre characteristics in salt tolerant and salt-sensitive lines of cotton (*Gossypium hirsutum L.*). Field Crops Res. 66, 115–127. doi: 10.1016/S0378-4290(00)00064-2

[B4] Bazihizina N. Barrett-Lennard E. G. Colmer T. D. (2012). Plant responses to heterogeneous salinity: growth of the halophyte Atriplex nummularia is determined by the root-weighted mean salinity of the root zone. J. Exp. Botany 63, 6347–635863. doi: 10.1093/jxb/ers302 23125356 PMC3504498

[B5] Bazihizina N. Colmer T. D. Barrett-Lennard E. G. (2009). Response to non-uniform salinity in the root zone of the halophyte Atriplex nummularia: growth, photosynthesis, water relations and tissue ion concentrations. Ann. Botany 104, 737–745. doi: 10.1093/aob/mcp151 19556265 PMC2729642

[B6] Feng Q. Gao Y. Li Y. F. Liu J.M. Gao F.K. Wang L.. (2022). The effects of water and salt stresses on growth, yield and quality of cotton in southern Xinjiang. J. Irrigation Drainage. 41, 73–81. doi: 10.13522/j.cnki.ggps.2022142

[B7] Fritschi F. B. Roberts B. A. Rains D. W. Travis R. L. Hutmacher R. B. (2004). Fate of nitrogen-15 applied to irrigated acala and pima cotton. Agron. J. 96, 646–655. doi: 10.2134/agronj2004.0646

[B8] Fu H. Y. Zhu Y. J. Wang Y. Q. (2013). Influence of drip irrigation with plastic mulch on soil salinity of cropland in Arid Regions. J. Irrigation Drainage. 32, 19–22. doi: 10.7631/j.issn.1672-3317

[B9] Guo Z. Lu Y. (2020). Study on the improvement of saline-alkali land by reed straw returning. Hans J. Agric. Sci. 10, 877–881. doi: 10.12677/HJAS.2020.1011134

[B10] Guo J. X. Lu X. Y. Tao Y. F. Guo H. J. Hou Z. N. Min W. (2022). Effects of saline and alkaline stresses on growth and nutrient uptake of cotton. Agric. Res. Arid. Areas. 40, 23–32. doi: 10.7606/j.issn.1000-7601

[B11] Kong X. Luo Z. Dong H. Z. Eneji A. E. Li W. J. (2012). Effects of non-uniform root zone salinity on water use, Na^+^ recirculation, and Na^+^ and H^+^ flux in cotton. J. Exp. Botany 63, 2105–2116. doi: 10.1093/jxb/err420 22200663 PMC3295398

[B12] Kong X. Luo Z. Dong H. Z. Eneji A. E. Li W. J. (2016). H_2_O_2_ and ABA signaling are responsible for the increased Na^+^ efflux and water uptake in *Gossypium hirsutum L.* roots in the non-saline side under non-uniform root zone salinity. J. Exp. Botany 67, 2247–2261. doi: 10.1093/jxb/erw026 26862153

[B13] Kong X. Luo Z. Dong H. Li W. J. Chen Y. (2017). Non-uniform salinity in the root zone alleviates salt damage by increasing sodium, water and nutrient transport genes expression in cotton. Sci. Rep. 1, 2879. doi: 10.1038/s41598-017-03302 PMC5460137 28588258

[B14] Loka D. A. Oosterhuis D. M. Ritchie G. L. (2011). Water-deficit stress in cotton. Stress Physiol. Cotton. 7, 37–72.

[B15] Lu D. Q. Wang Q. J. Wang W. Y. Shao M. A. (2001). Salt distribution and effect factors in under-film drip irrigation. Irrigation Drainage. 20, 28–31.

[B16] Luo Z. Hu Q. Y. Tang W. Wang X. W. Lu H. Q. Zhang Z.. (2022). Effects of N fertilizer rate and planting density on short-season cotton yield, N agronomic efficiency and soil N using ^15^N tracing technique. Eur. J. Agronomy 138, 126546. doi: 10.1016/j.eja.2022.126546

[B17] Luo Z. Kong X. Q. Dai J. L. Dong H. Z. (2015). Soil plus foliar Nitrogen application increases cotton growth and salinity tolerance. J. Plant Nutr. 38, 443–455. doi: 10.1080/01904167.2014.912324

[B18] Luo Z. Liu H. Li W. P. Zhao Q. Dai J. L. Tian. L. W.. (2018). Effects of reduced nitrogen rate on cotton yield and nitrogen use efficiency as mediated by application mode or plant density. Field Crops Res. 218, 150–157. doi: 10.1016/j.fcr.2018.01.003

[B19] Ma Z. B. Yan G. T. Liu G. Z. Huang Q. Li L. L. Zhu W. (2013). Effects of nitrogen application rates on main physiological characteristics of leaves, dry matter accumulation and yield of cotton cultivated in the Yellow River bottomlands. Plant Nutr. Fertilizer Sci. 19, 849–857. doi: 10.11674/zwyf.2013.0410

[B20] Maas E. V. Hoffman G. J. (1977). Crop salt tolerance-current assessment. J. Irrigation Drainage Division ASCE 103, 115–134. doi: 10.1061/JRCEA4.0001137

[B21] Mao S. C. (2013). Cultivation of Cotton in China (Shanghai, China: Shanghai Science and Technology Press).

[B22] Meng H. B. Jiang S. S. Hua S. J. Lin X. Y. Li Y. L. Guo W. L.. (2011). Comparison between a tetraploid turnip and its diploid progenitor (*Brassica rapa L.*): the adaptation to salinity stress. J. Integr. Agric. 10, 363–375. doi: 10.1016/S1671-2927(11)60015-1

[B23] Mu H. C. Tumaerbai. H. Su L. T. Mahemujiang A. Wang Y. M. Zhang I. Z.. (2011). Salt transfer law for cotton field with drip irrigation under mulch in arid region. Trans. CSAE 27, 18–22. doi: 10.3969/j.issn.1002-6819.2011.07.004

[B24] Munawar W. Hameed A. Khan M. (2021). Differential morpho physiological and biochemical responses of cotton genotypes under various salinity stress levels during early growth stage. Front. Plant Sci. 12. doi: 10.3389/fpls.2021.622309 PMC7990906 33777064

[B25] Pessarakli M. (2001). “Physiological responses of cotton (*Gossypium hirsutum L.*) to salt stress,” in Handbook of Plant and Crop Physiology (New York, USA: Marcel Dekker), 681–696.

[B26] Qi G. P. Zhang E. H. Zhang B. C. Li L. L. Qin S. H. (2007). Effects on maize root growth and soil water-salt content in root zone subjected to irrigation under different soil salt treatments. J. Desert Res. 6, 1007–1011.

[B27] Roberts T. L. Slaton N. A. Kelley J. P. Greub C. E. Fulford A. M. (2016). Fertilizer nitrogen recovery efficiency of furrow-irrigated corn. Agron. J. 108, 2123–2128. doi: 10.2134/agronj2016.02.0092

[B28] Rocha-Munive M. G. Soberon M. Castaneda S. Niaves E. Scheinvar E. Eguiarte L. E.. (2018). Evaluation of the impact of genetically modified cotton after 20 years of cultivation in Mexico. Front. Bioeng. Biotechnol. 6. doi: 10.3389/fbioe.2018.00082 PMC6023983 29988354

[B29] Shah A. N. Javed T. Singhal R. K. Shabbir R. Wang D. Hussain S.. (2022). Nitrogen use efficiency in cotton: Challenges and opportunities against environmental constraints. Front. Plant Sci. 13. doi: 10.3389/fpls.2022.970339 PMC9443504 36072312

[B30] Sharif I. Aleem S. Farooq J. Rizwan M. Chohan S. M. (2019). Salinity stress in cotton: effects, mechanism of tolerance and its management strategies. Physiol. Mol. Biol. Plants an Int. J. Funct. Plant Biol. 25, 807–820. doi: 10.1007/s12298-019-00676-2 PMC6656830 31402811

[B31] Siddiqui M. H. Mohammad F. Khan M. N. Al-Whaibi M. H. Bahkali A. H. (2010). Nitrogen in relation to photosynthetic capacity and accumulation of osmoprotectant and nutrients in Brassica genotypes grown under salt stress. Scientia Agricultura sinica. 9, 671–680. doi: 10.1016/S1671-2927(09)60142-5

[B32] Sikder R. K. Wang X. Zhang H. Gui H. Dong Q. Jin D.. (2020). Nitrogen enhances salt tolerance by modulating the antioxidant defense system and osmoregulation substance content in *Gossypium hirsutum* . Plants (Basel) 9, 450. doi: 10.3390/plants9040450 32260233 PMC7238023

[B33] Singh M. (2014). Plant tolerance mechanism against salt stress: The nutrient management approach. Biochem. Pharmacol. (Oxford) 3, e165. doi: 10.4172/2167-0501

[B34] Singh M. Singh V. P. Prasad S. M. (2019). Nitrogen alleviates salinity toxicity in Solanum lycopersicum seedlings by regulating ROS homeostasis. Plant Physiol. Biochem. 141, 466–476. doi: 10.1016/j.plaphy.2019.04.004 31252252

[B35] Tan J. Kang Y. Jiao Y. Sun Z. Weo L. Feng D.. (2008). Characteristics of soil salinity and salt ions distribution in salt-affected field under mulch-drip irrigation in different planting years. Trans. CSAE 24 (6), 59–63. doi: 10.3901/JME.2008.09.177

[B36] Tang Q. Y. Feng M. G. (2002). DPS Data Processing System for Practical Statistics (Beijing: Science Press).

[B37] Wang W. X. Vinocur B. Altman A. (2003). Plant responses to drought, salinity and extreme temperatures: towards genetic engineering for stress tolerance. Planta 21, 1–14. doi: 10.1007/s00425-003-1105-5 14513379

[B38] Wang Q. J. Wang W. Y. Lv D. Q. Wang Z. R. Zhang J. F. (2000). Water and salt transport features for salt-effected soil through drip irrigation under film. Trans. Chin. Soc. Agric. Eng. 16, 54–57.

[B39] Xue X. P. Guo W. Q. Wang Y. L. Zhang L. J. Zhou Z. G. (2006). Research on dynamic increase characteristics of dry matter of cotton at different nitrogen levels. Cotton Sci. 18, 323–326.

[B40] Yan K. F. Peng F. T. Qi Y. J. Dang Z. Q. Zhang J. H. Jiang X. M. (2015). Effects of different irrigation and fertilizer methods on space variation of nitrate nitrogen and nitrogen fertilization absorption and utilization of plants. Water Saving Irrig. 9, 33–38.

[B41] Ye X. Wang Y. D. Li R. X. (2004). Comparative studies of dry matter accumulation in different cotton varieties. J. Southwest Agric. Univ. 26, 749–752.

[B42] Yin H. H. Li Q. Z. Li H. T. Shang N. Zhang H. Li T.. (2016). Effect of different soil textures on cotton yield, composition factors and boll spatial-temporal distribution in western Shandong. Chin. Agric. Sci. Bull. 32, 87–90.

[B43] Zhang Z. Chattha M. S. Ahmed S. Liu J. H. Liu A. D. Yang L. R.. (2021). Nitrogen reduction in high plant density cotton is feasible due to quicker biomass accumulation. Ind. Crops Prod. 172, 114070. doi: 10.1016/j.indcrop.2021.114070

[B44] Zhang L. Ma H. Chen T. Pen J. Yu S. Zhao X. (2014). Morphological and physiological responses of cotton (*Gossypium hirsutum L.*) plants to salinity. PloS One 12, e112807. doi: 10.1371/journal.pone.0112807 PMC4229235 25391141

[B45] Zhang Y. Zhu Y. Yao B. (2020). A study on inter annual change features of soil salinity of cotton field with drip irrigation under mulch in Southern Xinjiang. PloS One 15, e0244404. doi: 10.1371/journal.pone.0244404 33378388 PMC7773193

[B46] Zheng J. C. Yan M. M. Zhang J. S. Gao L. L. Shi H. L. Zheng H.. (2016). Effects of nitrogen application on growth and nitrogen accumulation of cotton under shading condition. J. Plant Nutr. Fertilizers 22, 94–103.

[B47] Zhu Z. Z. Chen L. Yang G. Z. Song X. Z. Wang D. P. Chen Q. Z.. (2011). Study progress on dry matter and nutrient accumulation distribution of China cotton. Jiangxi Cotton. 33, 7–19.

