# Supplementary material for: Effects of N application methods on cotton yield and fertilizer N recovery efficiency in salinity fields with drip irrigation under mulch film using 15N tracing technique
Source: Front Plant Sci. 2024 Apr 26;15:1394285. doi: 10.3389/fpls.2024.1394285 (PMC11084282; doi:10.3389/fpls.2024.1394285)
Supplement: Supplementary file 1 [file DataSheet_1.pdf]

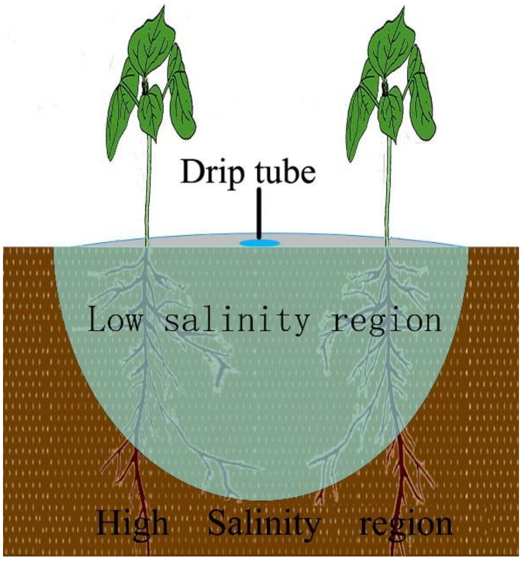

Supplemental Fig 1. The distribution of salt in soil on drip irrigation under mulch film condition.

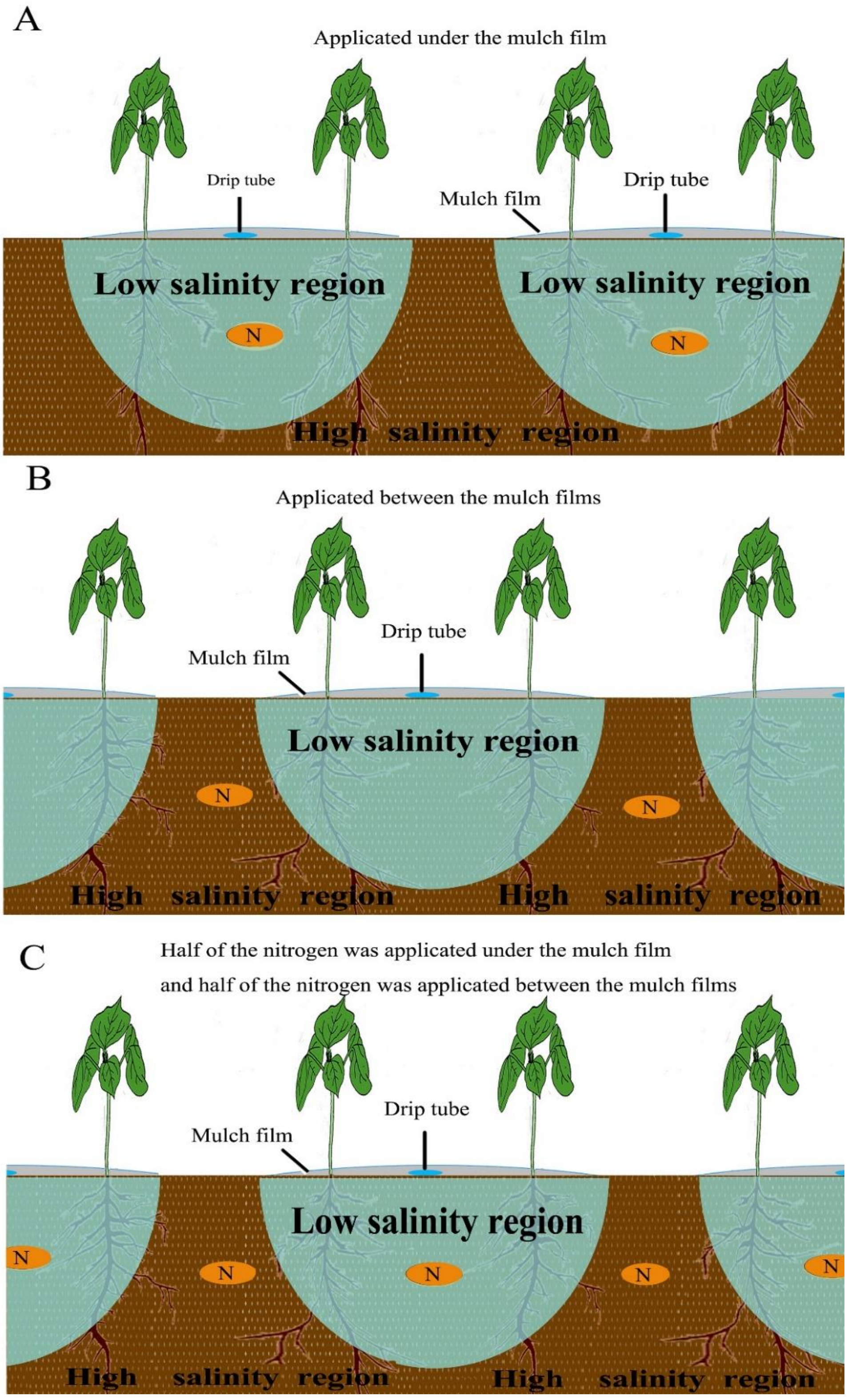

Supplemental Fig.2 Schematic diagram of different N application methods. UM (A), BM (B) and HUHB (C).

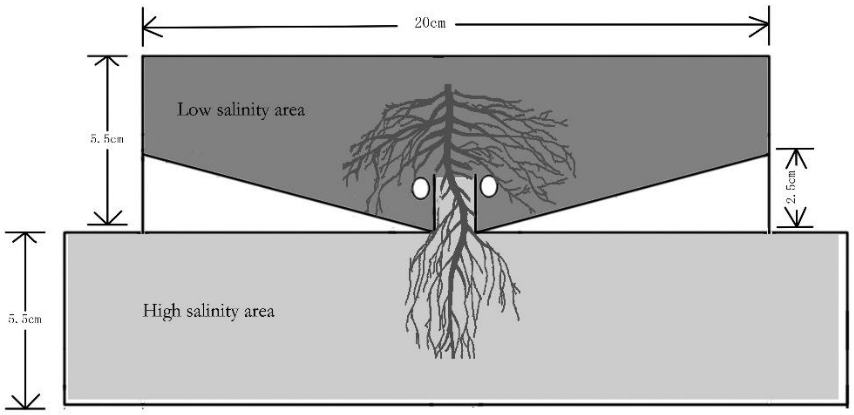

Supplemental Fig.3 Schematic diagram of longitudinal salt difference distribution device.

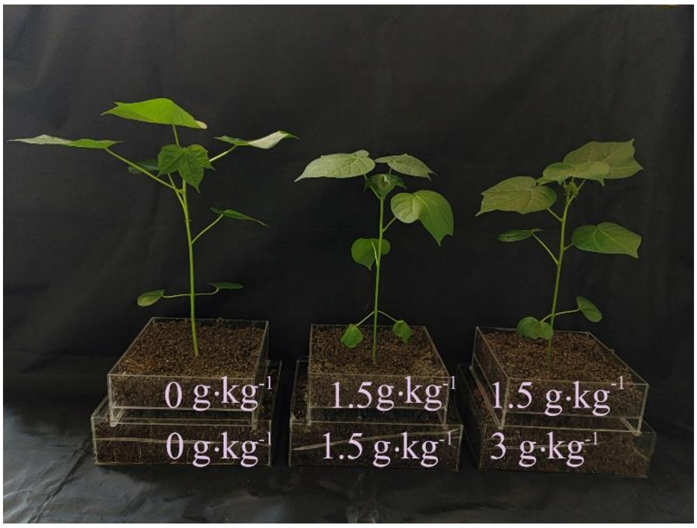

Supplemental Fig.4 The treatments with the different NaCl content (0, 1.5 and 3.0 $g{\cdot}kg^{-1}$) in upper or lower parts.

Supplemental Table 1 Primers used for qRT-PCR analysis

**Gene name**	**Forward primer (5’-3’)**	**Reverse primer (5’-3’)**
GhNRT1.1/Gh_A12G2001	TAGGGTTTTTCGTTAGTTCTGTGCT	TCCCTTCATCCGCAAGTCTCT
GhNRT2.1/Gh_D03G1307	GTAGCGAGATCAACCGAGGAAC	TGCAATATTTCATCTTGGGTTGG
GhNRT1.5/Gh_A08G1434	ATGTTGAAGAGCAGAAGAAGGGAA	GTCGAAGCTAGACATGCTGGCT
